# Front‐line treatment efficacy and clinical outcomes of elderly patients with multiple myeloma in a real‐world setting: A multicenter retrospective study in China

**DOI:** 10.1002/cam4.5234

**Published:** 2022-10-21

**Authors:** Li Bao, Ai‐Jun Liu, Bin Chu, Qian Wang, Yu‐Jun Dong, Min‐Qiu Lu, Lei Shi, Shan Gao, Yu‐Tong Wang, Li‐Fang Wang, Wen‐Ming Chen, Jun‐Ling Zhuang

**Affiliations:** ^1^ Department of Hematology Beijing Jishuitan Hospital Beijing China; ^2^ Department of Hematology Beijing Chaoyang Hospital Beijing China; ^3^ Department of Hematology Peking University First Hospital Beijing China; ^4^ Department of Epidemiology and Statistics Beijing Jishuitan Hospital Beijing China; ^5^ Department of Hematology Peking Union Medical College Hospital Beijing China

**Keywords:** front‐line treatment, immunomodulatory drugs, multiple myeloma, overall survival, proteasome inhibitors

## Abstract

**Background:**

The use of proteasome inhibitors (PIs), new immune modulators (IMiDs), and other new drugs, as well as high‐dose chemotherapy combined with autologous stem cell transplantation has considerably improved the survival of young patients with multiple myeloma (MM). However, the improvement in survival among elderly patients remains insufficient. Optimal treatment recommendation models for elderly patients with MM have not been developed especially there are quite few study in the real world.

**Methods:**

We retrospectively analyzed the treatment patterns and outcomes of 328 Chinese patients (≥65 years) with MM in a real‐world setting. Patients were divided into three groups according to induction regimens.

**Results:**

The median age of the cohort was 70 (65–86) years. The patients were divided into group 1 (PIs based regimens, *n* = 218), group 2 (IMiDs based regimens, *n* = 48) and group 3 (PIs + IMiDs, *n* = 62). Induction regimens in group 3 produced higher overall response rate than group 1 and 2 (85.42% vs. 71.08% vs. 66.67%, *p* = 0.016). The median follow‐up of the cohort was 30 (interquartile range [IQR] 18–36) months. For the entire cohort median progression‐free survival (PFS) was 26 (IQR 12.00–42.89) months and overall survival (OS) was 60 (IQR 40.00–67.20) months. The PFS were not significantly different among the three groups (28 months vs. 18 months vs. 26 months, *p* = 0.182). So were the OS (60 months vs. 59 months vs. not reached, *p* = 0.067). Multivariate analysis revealed that age >70 year, frailty status (Geriatric vulnerability score), induction efficacy < partial remission, and no maintenance treatment were independent poor prognostic factors for OS.

**Conclusion:**

Front‐line induction regimens combining PIs and IMiDs developed more deep response than single PI or IMiD based regimens. Maintenance treatment can further improve the clinical outcome in elderly MM patients in real‐world setting.

## Graphical abstract

Front‐line treatment for elderly MM patients.

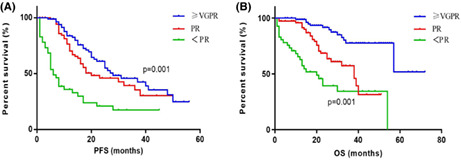

## 1 INTRODUCTION

Multiple myeloma (MM) is a malignant clonal plasma cell disorder that is currently considered incurable. The median age at diagnosis is approximately 70 years and the incidence increases with age.^1^, ^2^ The use of proteasome inhibitors (PIs), immune modulators (IMiDs), and other  novel drugs, as well as high‐dose chemotherapy combined with autologous stem cell transplantation, has considerably improved the survival of patients with MM. Although with continuing advances in treatments the survival rates are improving, age at diagnosis remains an important determinant of outcome.^3^

In general, patients with newly diagnosed MM are classified into two groups: transplant‐eligible and transplant‐ineligible and are treated by different strategies. For the transplant‐eligible patients a three‐drug combination induction therapy followed by consolidation with transplantation and then maintenance treatment have been considered as standard treatment pattern.^4^ For transplant‐ineligible patients, the treatment is widely heterogenous. The dividing line betwen induction and maintenance is not clear depending on physicians' discretion in different areas.

Over the past 20 years, treatment of transplant‐ineligible MM patients evolved from melphalan/prednisone to IMiD or PI based regimens, and then to a three‐drug combination based on backbone of lenalidomide/dexamethasone. More recently, anti‐CD38 mAb therapy has also been incorporated into these regimens. In addition, moving from fixed duration treatment to continuous therapy also contributed to the improved survival outcomes. Nevertheless, for elderly MM patients, the optimal model of treatment is not standardized yet.

So far, the therapeutic experience with MM has dominated by data from clinical trials in which the elderly patients were often excluded due to poor physical status and comorbidities. So the evidence of elderly patients from the real‐world practice is even more noteworthy. Up till now quite few relevant real‐world studies have been conducted on elderly subpopulation with MM, mostly from Europe and North America.^5^, ^6^, ^7^ There is a lack of data on treatment patterns and survival of elderly Asian patients. In China, novel agents such as bortezomib, ixazomib, and lenalidomide, have been reimbursed by medical insurance system after 2016, which brought advantages of clinical availability. Before that, the utilization of novel agents was only approximately 50%^8^. Therefore, dramatic changes could have developed during past 5 year. In this study, we analyzed the front‐line treatment models in elderly MM patients from four major medical centers, compared the impact of induction regimens and maintenance therapy on clincial outcomes, and discussed the current problems in real‐world practice, providing a reference to further improve the survival of elderly MM patients in the future.

## 2 MATERIALS AND METHODS

### 2.1 Study design and patients

This was a retrospective cohort study. Data of 362 newly diagnosed elderly MM patients (age ≥65 years) were collected from four medical centers in China between January 2016 and December 2020, and all patients met the International Myeloma Working Group (IMWG) diagnostic criteria.^9^ Patients with smoldering MM, plasma cell leukeima or solitary plasmacytoma were excluded. Only a few patients were treated with conventional chemotherapy so that this minor group was excluded. The four centers were Beijing Jishuitan Hospital, Beijing Chaoyang Hospital, Peking University First Hospital and Peking Union Medical College Hospital. The retrospective follow‐up period was till December 2021. The protocol was approved by the institutional medical ethics committee and informed consent were assigned before treatment (Ethical approval number JST201907‐04).

### 2.2 Research variables

For each patient, data regarding age, gender, type of M protein, Hemoglobin (HGB), calcium, dehydrogenase (lactate dehydrogenase [LDH]) and bone marrow plasma cell perccentage as well as complications were collected as base‐line characteristics. The albumin (ALB) and beta 2‐microglobulin (β2‐MG) were recorded for International Staging System (ISS). Estimated glomerular filtration rate (eGFR) was calculated. Geriatric vulnerability score (GA score)^10^ was used to evaluate the frailty status. High‐risk chromosomal abnormalities^11^ referred to any one of amplification of 1q21, 17P deletion, t (4;14),t (14;16), or t (14;20) detected by fluorescence in situ hybridization (FISH).

### 2.3 Treatment process

Patients were divided into three groups according to the front‐line induction therapies including PIs (bortezomib or ixazomib, group 1), regimens including IMiDs (lenalidomide or thalidomide, group 2), and regimens combining PIs and IMiDs (group 3). None of the patients underwent transplantation. The maintenance treatment after front‐line induction comprised IMiDs or PIs or both. The optimal response^12^ after induction was reflected in partial response (PR), very good partial response (VGPR) and complete response (CR). The overall response rate (ORR) = PR + VGPR+CR. Efficacy PR or better were regarded as effective. Patents whose response were less than PR were considered ineffective and administrated the second‐line regimens.

### 2.4 Follow‐up

Till December 2021, clinical outcomes were assessed using progression‐free survival (PFS) and overall survival (OS) with a median follow‐up of 30 months (interquartile range [IQR] 18–36). PFS refers to the time from the beginning of therapy to the first occurrence of disease progression or death. OS refers to the time from diagnosis to death, regardless of causes.

### 2.5 Statistical methods and data analyses

Patients were grouped by front‐line induction regimens. The clinical characteristics of our cohort were done using descriptive statistics and non‐normally distributed data in each group were analyzed using the Mann–Whitney *U* test. The comparison of interest defined a response priority between PIs + IMiDs regimen versus PIs and PIs + IMiDs versus IMiDs. Logistic regression was used to evaluate the association between induction response and the front‐line regimens (VGPR and better response vs. other). In order to control confounding factors, we conducted multivariable analysis adjusted for age, GA score, ALB, ISS stage, eGFR and FISH (results reported as adjusted OR and HR). PFS and OS were analyzed using Kaplan–Meier survival curves and the log‐rank test. Multivariate analysis of risk factors for PFS and OS was performed using Cox modeling. Two‐tailed *p* values <0.05 were considered as statistically significant. SPSS statistical software version 20.0 (IBM Corp.) was used in our analysis.

## 3 RESULTS

### 3.1 Patient baseline characteristics

#### 3.1.1 General description of the sample

Of the 362 patients, 24 (10 males and 14 females) abandoned treatment for economic reason, with a median age of 71 (65–85) years. Ten patients (six males and four females) treated with traditional chemotherapy was excluded. In total, 328 patients were included, and their baseline data were listed in Table 1. Briefly, there were 177 males and 151 females with median age of 70 (65–86) years. The detailed age distribution was 172 patients (52.43%) aged 65–70 years, 130 (39.63%) aged 71–79 years, and 26 (7.93%) aged ≥80 years. There were 59.76% of patients in ISS stage III and 36.89% in Revised International Scoring System (R‐ISS) stage III. FISH high‐risk percentage was 18.90% and GA score frail patients accounted for less than 1/3 (22.26%).

**TABLE 1:** Baseline data of newly diagnosed elderly MM patients (*n* = 328).

Parameter	Median value (range) or number (%)
Age, years old	
Median(range)	70 (65–86)
65–70	172 (52.44%)
71–79	130 (39.63%)
≥80	26 (7.93%)
Male	177 (53.96%)
Female	151 (46.04%)
M protein	
IgG	146 (44.51%)
IgA	77 (23.48%)
IgD	16 (4.88%)
Light chain	61 (18.60%)
Non‐secreting	18 (5.49%)
Missing	10 (3.04%)
DS stage	
I	17 (5.18%)
II	41 (12.50%)
III	264 (80.49%)
Missing	6 (1.83%)
SCr (mol/L)	86.20 (31–1370)
Ca (mmol/L)	2.32 (1.11–4.73)
HGB (g/L)	97 (42–158)
LDH (IU/L)	173 (14–2048)
β 2‐MG (mg/L)	5.58 (0.79–58.06)
ALB (g/L)	33.15 (12.50–47.90)
ISS stage	
I	36 (10.97%)
II	81 (24.70%)
III	196 (59.76%)
Missing	15 (4.57%)
R‐ISS stage	
I	24 (7.32%)
II	146 (44.51%)
III	121 (36.89%)
Missing	37 (11.28%)
BMPC percentage (%)	24.25 (0–98)
FISH	
Standard‐risk	203 (61.89%)
High‐risk	62 (18.90%)
Missing	63 (19.21%)
GA score	
Fit	114 (34.76%)
Intermediate fit	17 (5.18%)
Frail	73 (22.26%)
Missing	124 (37.80%)

Abbreviations: ALB, serum albumin; BMPC, bone marrow plasma cells; Ca, corrected serum calcium; FISH, fluorescence in situ hybridization; GA score, geriatric vulnerability score; HGB, hemoglobin; ISS, International Scoring System; LDH, lactate dehydrogenase; R‐ISS, Revised International Scoring System; SCr, serum creatinine; β2‐MG, beta 2‐microglobulin.

#### 3.1.2 Baseline difference between the three groups

As shown in Table 2, the proportions of hypoproteinemia (ALB <30 g/L), kidney failure (eGFR ≤40 ml/min), FISH high‐risk, or GA score indicating fitness in group 3 were higher than those in group 1 and 2 (*p* < 0.05). There were no significant differences in other characteristics including age, gender, β2‐MG levels, marrow plasma cell percentage and the proportion of hypercalcemia (≥2.75 mmol/L), high LDH (≥250 IU/L), anemia (HGB <100 g/L), and ISS or R‐ISS stage distribution among the three groups (*p* > 0.05).

**TABLE 2:** Comparison of clinical characteristics of patients in the three groups receiving different front‐line induction regimens.

	Total (*n* = 328)	PIs (Group 1, *n* = 218)	IMiDs (Group 2, *n* = 48)	PIs + IMiDs (Group 3, *n* = 62)	*p* value
Age (year)	70 (65–86)	70 (65–86)	72 (65–86)	69 (65–82)	0.068
Male	177 (53.96%)	122 (55.96%)	23 (47.92%)	32 (51.61%)	0.502
Female	151 (46.04%)	96 (44.04%)	25 (52.08%)	30 (48.39%)	
Calcium ≥2.75 mmol/L	36 (10.98%)	29 (13.30%)	3 (6.25%)	4 (6.45%)	0.196
eGFR ≤40 ml/min	64 (19.51%)	41 (18.80%)	7 (14.58%)	16 (25.80%)	0.043
LDH ≥250 IU/L	51 (15.55%)	37 (16.97%)	5 (10.42%)	9 (14.52%)	0.355
HGB <100 g/L	187 (57.01%)	128 (58.72%)	26 (54.17%)	33 (53.23%)	0.831
β2‐MG (mg/L)	5.58 (0.79–58.06)	5.51 (1.18–58.06)	5.01 (2.10–51.00)	5.76 (0.79–39.93)	0.618
ALB <30 (g/L)	86 (26.22%)	48 (22.02%)	10 (20.83%)	28 (45.16%)	0.003
BMPC (%)	24.25 (0–98)	23.75 (0–98)	29.50 (0–88)	28.50 (0–93)	0.115
ISS stage					
I	36 (10.96%)	25 (11.47%)	7 (14.58%)	4 (6.45%)	0.191
II	81 (24.70%)	57 (26.15%)	12 (25.00%)	12 (19.35%)	
III	196 (59.76%)	128 (58.71%)	26 (54.17%)	42 (67.74%)	
Missing	15 (4.57%)	8 (3.67%)	3 (6.25%)	4 (6.45%)	
R‐ISS stage					0.147
I	24 (7.32%)	17 (7.80%)	6 (12.50%)	1 (1.60%)	
II	146 (44.51%)	97 (44.50%)	22 (45.80%)	27 (43.50%)	
III	121 (36.89%)	80 (36.70%)	14 (29.20%)	27 (43.50%)	
Missing	37 (11.28%)	24 (11.00%)	6 (12.50%)	7 (11.30%)	
GA score					0.001
Fit	114 (34.76%)	64 (29.36%)	12 (25.00%)	38 (61.29%)	
Intermediate‐fit	17 (5.18%)	13 (5.96%)	1 (2.08%)	3 (4.84%)	
Frail	73 (22.26%)	65 (29.82%)	5 (10.42%)	3 (4.84%)	
Missing	124 (37.80%)	76 (34.86%)	30 (62.50%)	18 (29.03%)	
FISH					0.044
Standard‐risk	203 (61.89%)	140 (64.20%)	30 (62.50%)	33 (53.20%)	
High‐risk	62 (18.90%)	36 (16.51%)	8 (16.70%)	18 (29.00%)	
Missing	63 (19.21%)	42 (19.27%)	10 (20.80%)	11 (17.70%)	

Abbreviations: ALB, albumin; eGFR, estimated glomerular filtration rate; FISH, fluorescence in situ hybridization; GA score, geriatric vulnerability score; LDH, lactic dehydrogenase; β2‐MG, beta 2 microglobulin.

### 3.2 Front‐line induction regimens and efficacy

Of all the 328 patients, 75 patients who did not complete two cycles of treatment were not applicable for response evaluation. In total, 253 patients received a median 8 (2–12) cycles of induction chemotherapy. There as no differences among the three groups in terms of median front‐line cycles (eight cycles in group 1 and 3, nine in group 2, *p* = 0.133). There were 185 patients whose response were PR or better and the total ORR was 73.12% (185/253). The ORRs of groups 1, 2, and 3 were 71.08%, 66.67%, and 85.42%, respectively. The proportion of patients in each group who achieved deep response (VGPR or better, ≥VGPR) was 39.16%, 25.64% and 62.50%, respectively. The ORR and deep response rates of group 3 were higher than group 1 and 2 (*p* = 0.016 and *p* = 0.018) (Table 3). Multivariable analysis controlling for age, GA score, eGFR, ALB, ISS and FISH risk confirmed an independent effect of the type of front‐line inducion regimens administered in the odds of achieving VGPR or better. PIs + IMiDs regimens represented deeper response compared with PIs or IMiDs only regimen (adjusted OR, PIs + IMiDs vs. PIs = 0.33; 95% CI 0.13–0.96, *p* = 0.042; adjusted OR, PIs + IMiDs vs. IMiDs = 0.29; 95% CI 0.11–0.72, *p* = 0.043) (Table 4).

**TABLE 3:** Induction treatment effect in the three groups receiving different front‐line induction regimens.

Induction treatment effect	Total (*n* = 253)	PIs (Group 1, *n* = 166)	IMiDs (Group 2, *n* = 39)	PIs + IMiDs (Group 3, *n* = 48)	*p* value
ORR	185 (73.12%)	118 (71.08%)	26 (66.67%)	41 (85.42%)	0.016
CR + VGPR	105 (41.50%)	65 (39.16%)	10 (25.64%)	30 (62.50%)	0.018
PR	80 (31.62%)	53 (31.92%)	16 (41.03%)	11 (22.92%)	0.069
<PR	68 (26.88%)	48 (28.92%)	13 (33.33%)	7 (14.58%)	0.013

Abbreviations: CR, complete response; IMiDs, immune modulators; ORR, overall response rate; PIs, proteasome inhibitors; PR, partial response; VGPR, very good partial response.

**TABLE 4:** Association between the front‐line regimens and response rate controlling for age, GA score, ISS stage, eGFR, ALB and FISH risk.

	Adjusted OR	95% CI	*p* value
Front‐line regimens			
PIs + IMiDs	1		
PIs	0.33	0.13–0.96	0.042
IMiDs	0.29	0.11–0.72	0.043
Age at diagnosis			
65–70	1		
71–79	0.65	0.04–2.73	0.842
≥80	0.79	0.11–1.01	0.714
GA score			
Fit	1		
Intermediate fit	1.38	0.69–4.67	0.181
Frail	1.07	0.32–3.49	0.127
ISS stage			
I/II	1		
III	1.42	0.21–2.99	0.998
eGFR			
≥40 ml/min	1		
<40 ml/min	1.30	0.42–2.37	0.638
ALB			
≥30 g/L	1		
<30 g/L	1.01	0.47–1.64	0.073
FISH			
Standard‐risk	1		
High‐risk	0.98	0.16–2.37	0.60

Abbreviations: ALB, serum albumin; eGFR, estimated glomerular filtration rate; FISH, fluorescence in situ hybridization; GA score, geriatric vulnerability score; ISS, International Scoring System.

### 3.3 Front‐line maintenance treatment

Among 185 patients whose response to induction therapy was PR or better, 129 continued maintenance therapy, while the other 56 withdrew further treatment. The median duration of maintenance was 26 (IQR 16–30) months, including 92 patients receiving IMiDs (73 lenalidomide and 19 thalidomide), 20 with PIs (16 ixazomib and 4 bortezomib), and 17 with PI + IMiDs (ixazomib + lenalidomide). During the course of maintenance treatment, the most common reasons of drug discontinuation were hematologic toxicity, rash, and diarrhea, however it was difficult to determine the specific incidence rate due to less detailed records.

### 3.4 Long‐term survival analysis

#### 3.4.1 The results of the univariate and multivariate analysis

In this study, there were 21 patients missing follow‐up and 307 patients were available for survival analysis. Of these, 66 died and 241 survived. The median PFS and OS of the entire cohort were 26 (IQR 12.00–42.8) months and 60 (IQR 40.00–67.20) months.

As shown in Table 5, univariate analysis revealed that age >70 years, GA score indicating frailness, R‐ISS stage III, high LDH, hypercalcemia, induction therapy efficacy < PR, and no maintenance were  unfavorable predictive factors of PFS (*p* < 0.05). Age >70 years, GA score of frailty, R‐ISS stage III, high LDH, hypercalcemia, eGFR ≤40 ml/min, high‐risk FISH, and no maintenance were risk factors predicting OS (*p* < 0.05). Yet, multivariate analysis revealed that only GA score of frailty, induction therapy effect < PR, and no maintenance were independent prognostic factors of poor PFS (*p* < 0.05), and age >70 years, GA score of frailty, initial response worse than PR, and no maintenance were independent prognostic factors predicting poor OS (*p* < 0.05).

**TABLE 5:** Risk factors for PFS and OS by multivariate analyses.

Risk factors for PFS	Univariate analysis		Multivariate analyses	
	HR	*p*	HR	*p*
Age >70 year old	1.379 (1.022–1.862)	**0.036**	1.084 (0.696–1.688)	0.722
Hypercalcemia (≥2.75 mmol/L)	1.916 (1.031–2.980)	**0.004**	1.537 (0.841–2.808)	0.162
eGFR≤40 mL/min	1.213 (0.846–1.741)	0.294		
High LDH ≥250 IU/L	1.671 (1.145–2.439)	**0.008**	1.503 (0.746–3.027)	0.254
ISS stage III	0.949 (0.693–1.299)	0.743		
RISS stage III	1.675 (1.200–2.339)	**0.002**	0.986 (0.558–1.741)	0.961
FISH high‐risk	1.236 (0.856–1.785)	0.258		
GA score frail	1.906 (1.101–1.891)	**0.008**	1.998 (1.284–3.110)	**0.002**
Induction efficacy < PR	3.227 (2.125–4.900)	**0.001**	2.423 (1.424–4.122)	**0.001**
No maintenance treatment	1.961 (1.255–3.066)	**0.003**	4.689 (3.226–10.032)	**0.001**

Risk factors for OS	Univariate analysis		Multivariate analyses	
	HR	*p*	HR	*p*
Age >70 year old	1.938 (1.313–2.861)	**0.001**	2.303 (1.194–3.245)	**0.045**
Hypercalcemia (≥2.75 mmol/L)	2.771 (1.653–4.643)	**0.001**	2.113 (0/900–4.963)	0.086
eGFR ≤40 mL/min	1.811 (1.184–2.769)	**0.006**	1.026 (0.459–2.296)	0.949
High LDH ≥250 IU/L	1.895 (1.187–3.024)	**0.007**	2.198 (0.522–9.259)	0.283
ISS stage III	1.023 (0.684–1.531)	0.911		
RISS stage III	2.109 (1.354–3.284)	**0.001**	1.233 (0.811–2.788)	0.554
FISH high‐risk	1.598 (1.003–2.547)	**0.048**	1.647 (0.450–6.033)	0.451
GA score frail	2.173 (1.298–3.636)	**0.003**	2.858 (1.434–5.694)	**0.003**
Induction efficacy < PR	3.869 (2.339–6.400)	**0.001**	3.087 (1.532–6.220)	**0.002**
No maintenance treatment	2.305 (1.315–4.042)	**0.004**	2.809 (1.506–5.376)	**0.001**

*Note*: Bold indicates values less than 0.05.

Abbreviations: eGFR, estimated glomerular filtration rate; FISH, fluorescence in situ hybridization; GA score, geriatric vulnerability score; ISS, International Scoring System; LDH, lactate dehydrogenase; OS, overall survival; PFS, progression‐free survival; PR, partial response; R‐ISS, Revised International Scoring System.

#### 3.4.2 The effect of maintenance treatment on survivability

**FIGURE 1:**
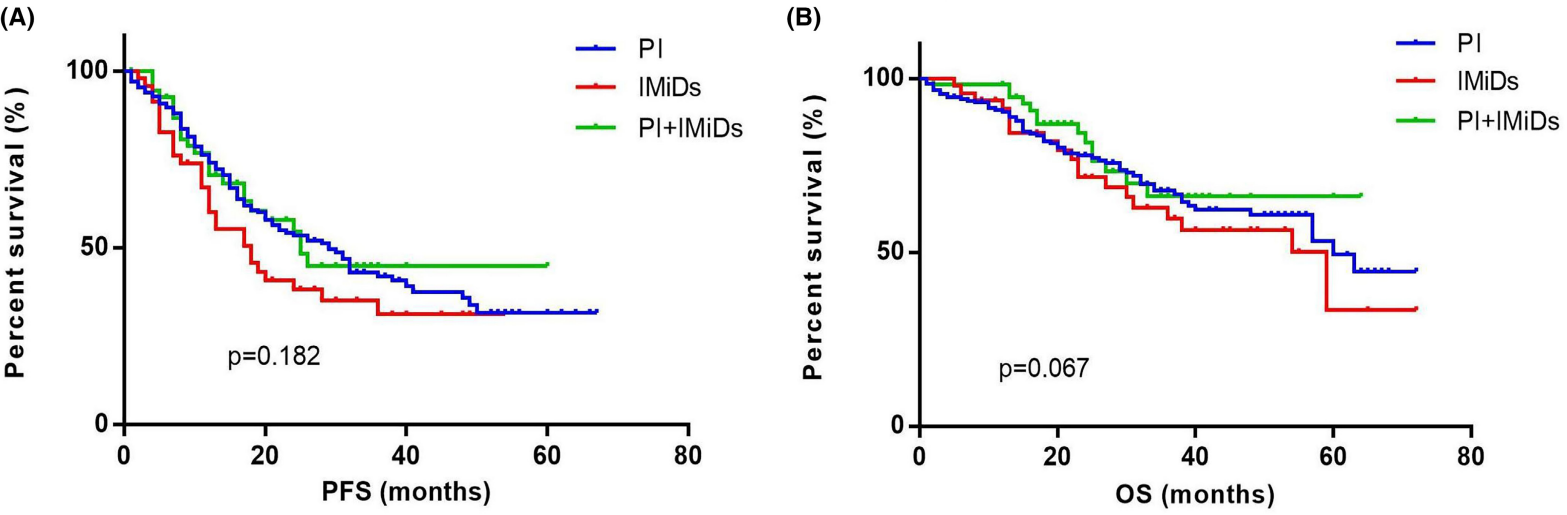
The survival curves for elderly patients with MM treated with different front‐line induction regimens. (A) The median PFS of patients in groups 1 (*n* = 219, PIs), group 2 (*n* = 42, IMiDs) and group 3 (*n* = 68, PIs + IMiDs) were 28, 18 and 26 months. There were no significant differences in PFS among the three groups (*p* = 0.182). (B) The median OS of patients in groups 1, 2, 3 were 60 months, 59 months and not reached. No significant differences were seen in the OS among the three groups (*p* = 0.067). IMiDs, immune modulators; MM, multiple myeloma; OS, overall survival; PFS, progression‐free survival; PI, proteasome inhibitor

The PFS in group 1, 2, 3 were 28 (IQR 13.00–39.25) months, 18 (IQR 11.00–22.00) months and 26 (IQR 12.00–33.00) months respectively. There were no significant differences in PFS among the three groups (*p* = 0.182). The OS in group 1, 2, 3 was 60 (IQR 29.00–71.00) months, 59 (IQR 23.00–69.00) months and not reached (IQR 25.00–NR) respectively (*p* = 0.067) (Figure 1).

**FIGURE 2:**
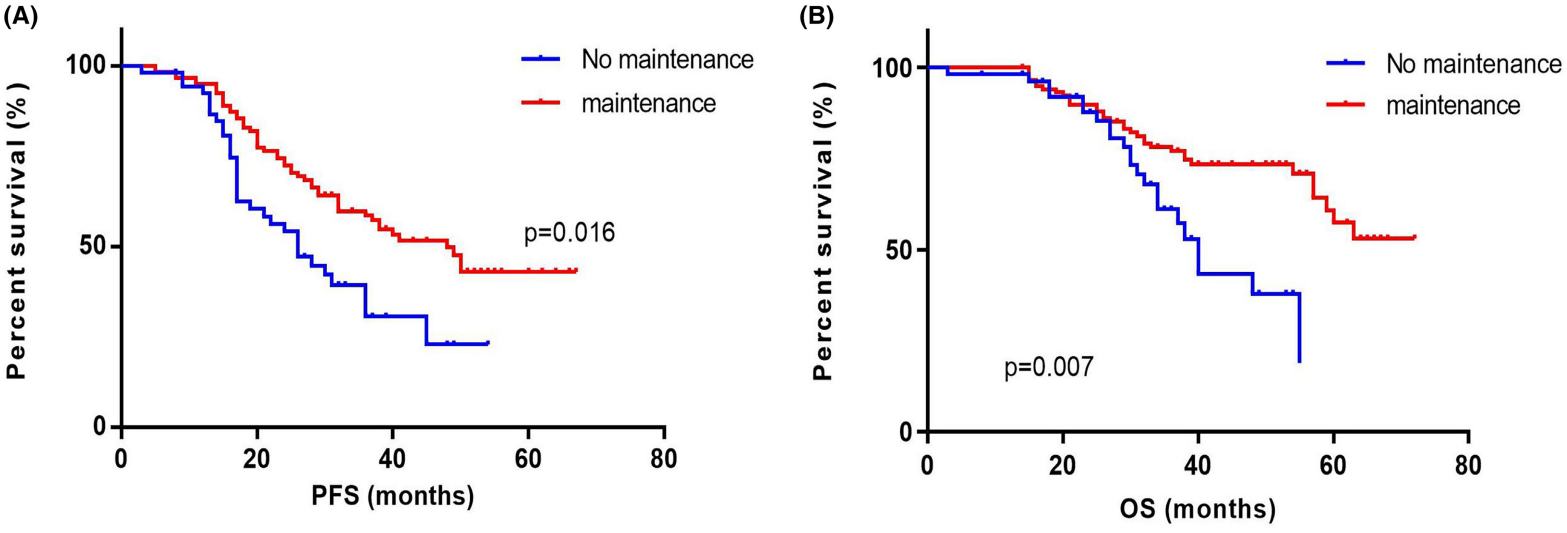
The survival curves for elderly patients in different maintenance treatment groups. (A) The median PFS of patients (*n* = 56) who did not receive maintenance therapy was 26 months. The median PFS of patients (*n* = 129) who received maintenance therapy was 48 months. There was significant difference in PFS between the two groups (*p* = 0.016). (B) The median OS of patients who did not receive maintenance therapy was 40 month. The median OS of patients who received maintenance therapy was not reached. There was significant difference in OS between the two groups (*p* = 0.007). OS, overall survival; PFS, progression‐free survival

The PFS and OS of patients (*n* = 56) who did not receive maintenance therapy was 26 (IQR 16.00–36.00) months and 40 (IQR 30.00–49.00) months. The PFS and OS of patients (*n* = 129) who received maintenance therapy was 48 (IQR 23.00–63.00) months and not reached (IQR 38.00–NR). The PFS and OS of patients did not receive maintenance therapy were both significantly shorter than those who did (*p* = 0.016 and *p* = 0.007) (Figure 2).

#### 3.4.3 The effect of age on survivability

**FIGURE 3:**
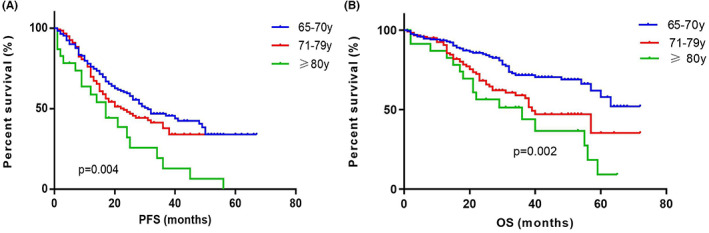
The survival curves for patients with MM across different age groups. (A) The median PFS of patients aged 65–70 years (*n* = 166), 71–79 years (*n* = 117) and ≥80 years (*n* = 24) were 31, 23 and 14 months. The PFS showed significant differences among the three different age groups (*p* = 0.004). (B) The median OS of patients aged 65–70, 71–79 and ≥80 years were not reached, 39 months and 37 months. The OS in age 65–70 year group was significantly longer than those in the other two groups (*p* = 0.002). MM, multiple myeloma; OS, overall survival; PFS, progression‐free survival

The PFS of patients aged 65–70 years (*n* = 166), 71–79 years (*n* = 117), and ≥80 years (*n* = 24) were 31 (IQR 14.00–45.20) months, 23 (IQR 12.00–40.00) months and 14 (IQR 7.00–24.00) months. The OS were not reached (IQR 32.00–NR), 39 (IQR 21.00–62.00) months and 37 (IQR 21.00–59.00) months. The PFS showed significant differences among different age groups (*p* = 0.004). The OS in age 65–70 group was significantly longer than those in age 71–79 and ≥80 groups (*n* = 0.002) but no differences were observed in OS between age 71 and 79 group and ≥80 group (*p* = 0.137) (Figure 3).

#### 3.4.4 The effect of induction response on survivability

**FIGURE 4:**
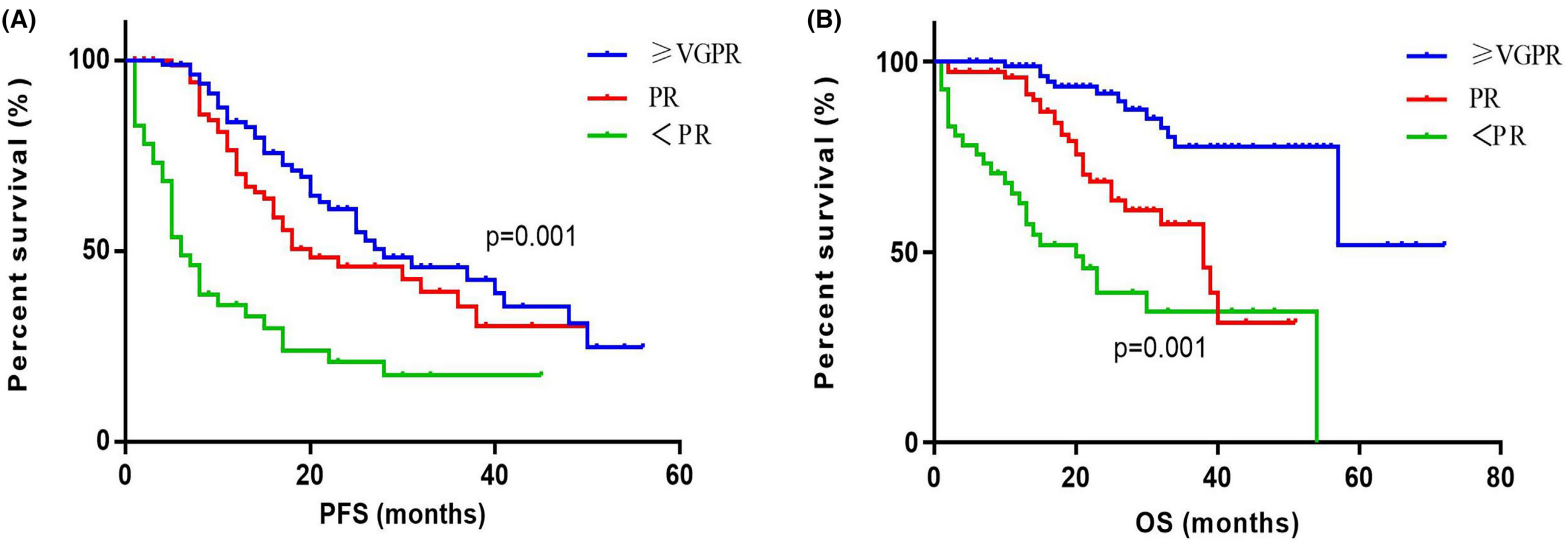
The survival curves for elderly patients in different induction response groups. (A) The median PFS of patients with responses ≥VGPR (*n* = 105), PR (*n* = 80) and <PR (*n* = 68) were 28, 20 and 6 months. The PFS showed significant differences among the three groups (*p* = 0.001). (B) The median OS of patients with responses ≥VGPR, PR and <PR were not reached, 38 and 20 months. The OS showed significant differences among the three groups (*p* = 0.001). OS, overall survival; PFS, progression‐free survival; VGPR, very good partial response

The PFS of patients with different responses (≥VGPR [*n* = 105], PR [*n* = 80], and <PR [*n* = 68]) were 28 (IQR 17.00–48.00) months, 20 (IQR 12.00–33.00) months, and 6 (IQR 4.00–22.00) months. The PFS showed significant differences among different response groups (*p* = 0.001). The corresponding OS of the three groups were not reached (IQR 57.00–NR), 38 (IQR 20.00–62.00) months, and 20 (IQR 8.00–54.00) months. The OS showed significant differences among different response groups (*p* = 0.001) (Figure 4).

#### 3.4.5 The effect of GA score on survivability

**FIGURE 5:**
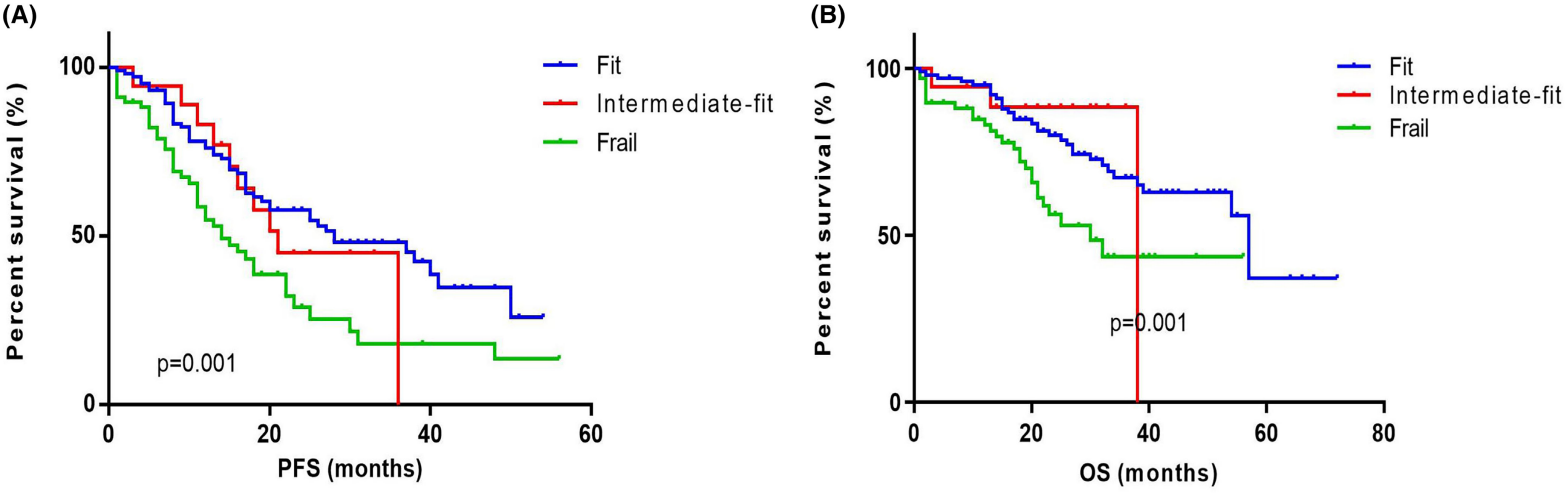
Survival curves for elderly patients with MM with different GA scores. The patients with GA scores (*n* = 204) were classified into fit group (0 score, *n* = 114), intermediate fit group (1 score, *n* = 17) and frail group (≥2 score, *n* = 73). (A) The median PFS in fit, intermediate fit and frail group were 28, 21 and 14 months. The PFS of the frail and intermediate fit groups were both significantly shorter than that of fit group (*p* = 0.001). (B) The median OS in fit, intermediate fit and frail group were 57, 38 and 30 months. The OS of the frail and intermediate fit groups were significantly shorter than that of fit group (*p* = 0.001). OS, overall survival; PFS, progression‐free survival

The patients with GA scores (*n* = 204) were classified into fit (0 score, *n* = 114, 55.88%), intermediate fit (1 score, *n* = 17, 8.33%), and frail (≥2 score, *n* = 73, 35.78%). The PFS were 28 (IQR 13.00–39.50) months, 21 (IQR 15.00–36.00) months and 14 (IQR 8.00–30.00) months. The OS were 57 (IQR 27.00–59.00) months, 38 (IQR 36.00–39.00) months and 30 (IQR 18.00–49.00) months. The PFS and the OS of the frail and intermediate fit groups were both significantly shorter than that of fit group (*p* = 0.001 and *p* = 0.001) (Figure 5).

## 4 DISCUSSION

In Chinese current clinical practice, MM patients aged ≥65 years are defined as elderly and considered ineligible for transplantation. Herein, we analyzed the treatment patterns of elderly MM patients from four big myeloma centers in China over the last 5 years. Our study suggests that even novel therapies including PIs and IMiDS are commonly administrated, age >70, general performance frailty, induction response < PR and no maintenance treatment are independent risk factors for OS.

Our data demonstrated distribution of front‐line regimens in elderly MM patients in multiple centers of China. Due to quick approval and reimbursement of medical insurance during past 5 years, PIs and IMiDs based regimens were prefered in front‐line treatment. In this study no patient received anti‐CD38 daratumumab because it was not approved as front‐line therapy in China until 2021.

Current treatment options for transplant‐ineligible MM patients are not unique, and clinical trials have shown that regimens containing two kinds of new drugs can further improve ORR and PFS compared with regimens containing only one kind of new drug.^13^, ^14^, ^15^ In these clinical tirals, the addition of anti‐CD38 daratumumab on the basis of bortezomib, melphalan and prednisone (Dara‐VMP) or lenalidomide and dexamethasone (Dara‐Rd) resulted in ORR over 90% and even longer PFS and OS. In our study we found that in real world PIs combined with IMiDs regimens produced the comparable ORR but the OS was not as good as clinical trials. In this study there were no differences in OS among patients treated with different first‐line induction regimen, which is different from previous real‐world reports.^5^, ^6^ We supposed it may be related to the regimen heterogeneity in different studies. Considering the clinical benefit of Dara‐Rd in transplant‐ineligible MM patients regardless of frailty status in clinical trial, it is worthy to explore the combination of daratumumab to the PIs or IMiDs regimens in elderly patients for further survival improvement in real world .

Maintenance treatment had a particular impact on the survival of elderly MM patients as response was further deepened.^16^ Our real world study also confirmed that the maintenance treatment after obtaining the response prolonged the PFS and OS in elderly MM patients. It is worth noting that about 30% (56/185) of the responders did not start first‐line maintenance treatment at the end of induction therapy. The original record did not tell us why these 56 patients refuse maintenance. But these data showed that elderly MM patients are not adequately treated in China. There were urgent need to improve this aspect.

In this study we found a significant correlation between first‐line induction response depth and survival. Similarly, the SWOGS0777 trial showed the deeper response of induction treatment represented the longer PFS and OS.^13^ Also the FIRST trial analysis showed 4‐year survival rates was closely related to the depth of induction response.^16^ Different from the above, a meta‐analysis suggested achieving CR was an independent predictor of long‐term outcome regardless of age and ISS stage.^17^ Anyhow in the new drug era, to pursue deep response in elderly patients is possible and still valuable. It is not only the induction regimens but the front‐line response that matters.

For patients with MM, various studies suggest that age is an important prognostic factor. In the era of traditional chemotherapy, the median OS in elderly MM population including 60–69, 70–79, and ≥80 years groups were only 20, 12, and 6 months, respectively.^18^ After novel agents were widely used, OS in corresponding age groups were remarkably prolonged in the real practice such as 22 months in patients over 80 years.^5^ This current study also showed that the older age means worse survival, but new drugs has significantly improved the outcome of elderly patients in different age groups especially the OS in ≥80 years group reached 37 months.

Due to comorbidities as well as the poor physical performance, a considerable part of elderly patients were excluded from clinical trials. Actually the assessment of frailty is very important because this can affect not only treatment choices but also clinical outcomes. In clinical trials, the GA score was found to be an independent predictor of OS, regardless of the patients' ISS stage, cytogenetic abnormalities, or treatment regimen.^10^ The European Myeloma Network proposed that elderly MM patients should adopt different treatment intensities according to their fitness,^19^ but currently the GA score is not widely used in the real world. The proportion of frail patients in our study was 35.78% (73/204) which was close to the report by Li et al.^20^ Our data suggested that GA score indicating fitness was independently correlated with longer OS, which was consistent with the conclusions in clinical trial.^10^ In a retrospective study all the patients were treated with bortezomib‐based regimen and the frail patients had shorter OS compared with fit and moderate‐fit patients (49 months vs. 63 months vs.63 months, *p* < 0.05).^21^ Together, these data verify the prognostic value of GA score in real practice.

In this study, factors such as high‐risk cytogenetic abnormalities and R‐ISS stage III, which were assumed to predict poor OS, did not show independent prognostic value. A recent report on clinical trial data indicated that the relative contribution of different prognostic factors in predicting disease risk varies according to age, and for older MM patients, frailty score and comorbidity assessment are more important than FISH and ISS stage.^22^ Considering that the prognostic value of these factors was mainly established through clinical trials, their value in the real world may require further research.

This study had some limitations. First, this was a retrospective study with unmatched clinical characteristics at baseline in different treatment regimens. But after we adjusted the confounding factors, PIs + IMiDs combination regimens still showed superior response. Second, adverse events and toxicity were not recorded detailed and absence of this aspect was a pity.

In summary, our cohort represented the real‐world treatment situations in elderly MM patients in China. Induction regimens containing PIs + IMiDs produced deeper overall response rate than only PIs or IMiDs based regimens and should be recommended to suitable patients. The maintenance treatment can further improve the outcome and this aspect needs our special attention.

## AUTHOR CONTRIBUTIONS

**Li Bao:** Writing – original draft (equal). **Ai‐Jun Liu:** Writing – original draft (equal). **Bin Chu:** Resources (equal). **Qian Wang:** Resources (equal). **Yu‐Jun Dong:** Resources (equal). **Min‐Qiu Lu:** Resources (equal). **Lei Shi:** Resources (equal). **Shan Gao:** Resources (equal). **Yu‐Tong Wang:** Resources (equal). **Li‐Fang Wang:** Formal analysis (equal). **Weng‐Ming Chen:** Resources (equal); supervision (equal). **Jun‐Ling Zhuang:** Conceptualization (equal); writing – review and editing (equal).

## CONFLICT OF INTEREST

The authors have stated that they have no conflicts of interest.

## Data Availability

The processed data required to reproduce these findings cannot be shared at this time as the data also is a part of an ongoing study.

## References

[1] Ferlay J , Soerjomataram I , Dikshit R , et al. Cancer incidence and mortality worldwide: sources, methods and major patterns in GLOBOCAN 2012. Int J Cancer. 2015;136(5):E359‐E386. 25220842 10.1002/ijc.29210

[2] Ferlay J , Steliarova‐Foucher E , Lortet‐Tieulent J , et al. Cancer incidence and mortality patterns in Europe: estimates for 40 countries in 2012. Eur J Cancer. 2013;49(6):1374‐1403. 23485231 10.1016/j.ejca.2012.12.027

[3] Costa LJ , Brill IK , Omel J , Godby K , Kumar SK , Brown EE . Recent trends in multiple myeloma incidence and survival by age, race, and ethnicity in the United States. Blood Adv. 2017;1(4):282‐287. 29296944 10.1182/bloodadvances.2016002493 PMC5727774

[4] Callander NS , Baljevic M , Adekola K , et al. NCCN guidelines(R) insights: multiple myeloma, version 3.2022. J Natl Compr Canc Netw. 2022;20(1):8‐19. 34991075 10.6004/jnccn.2022.0002

[5] Cejalvo MJ , Bustamante G , Gonzalez E , et al. Treatment patterns and outcomes in real‐world transplant‐ineligible patients newly diagnosed with multiple myeloma. Ann Hematol. 2021;100(7):1769‐1778. 33885924 10.1007/s00277-021-04529-5

[6] Joao C , Bergantim R , Neves M , et al. Multiple myeloma in elderly patients‐a Portuguese multicentric real‐life study. Ann Hematol. 2019;98(7):1689‐1701. 30963200 10.1007/s00277-019-03640-y

[7] Fiala MA , Foley NC , Zweegman S , Vij R , Wildes TM . The characteristics, treatment patterns, and outcomes of older adults aged 80 and over with multiple myeloma. J Geriatr Oncol. 2020;11(8):1274‐1278. 32169544 10.1016/j.jgo.2020.03.005 PMC7483605

[8] Qian X , Chen H , Xia J , Wang J , Zhou X , Guo H . Real‐world clinical outcomes in elderly Chinese patients with multiple myeloma: a single‐center experience. Med Sci Monit. 2018;23(24):5887‐5893. 10.12659/MSM.907588 PMC6118163 30138301

[9] Rajkumar SV , Dimopoulos MA , Palumbo A , et al. International myeloma working group updated criteria for the diagnosis of multiple myeloma. Lancet Oncol. 2014;15(12):e538‐e548. 25439696 10.1016/S1470-2045(14)70442-5

[10] Palumbo A , Bringhen S , Mateos MV , et al. Geriatric assessment predicts survival and toxicities in elderly myeloma patients: an international myeloma working group report. Blood. 2015;125(13):2068‐2074. 25628469 10.1182/blood-2014-12-615187 PMC4375104

[11] Sonneveld P , Avet‐Loiseau H , Lonial S , et al. Treatment of multiple myeloma with high‐risk cytogenetics: a consensus of the international myeloma working group. Blood. 2016;127(24):2955‐2962. 27002115 10.1182/blood-2016-01-631200 PMC4920674

[12] Palumbo A , Rajkumar SV , San Miguel JF , et al. International myeloma working group consensus statement for the management, treatment, and supportive care of patients with myeloma not eligible for standard autologous stem‐cell transplantation. J Clin Oncol. 2014;32(6):587‐600. 24419113 10.1200/JCO.2013.48.7934 PMC3918540

[13] Durie BGM , Hoering A , Abidi MH , et al. Bortezomib with lenalidomide and dexamethasone versus lenalidomide and dexamethasone alone in patients with newly diagnosed myeloma without intent for immediate autologous stem‐cell transplant (SWOG S0777): a randomised, open‐label, phase 3 trial. Lancet. 2017;389(10068):519‐527. 28017406 10.1016/S0140-6736(16)31594-X PMC5546834

[14] Mateos MV , Cavo M , Blade J , et al. Overall survival with daratumumab, bortezomib, melphalan, and prednisone in newly diagnosed multiple myeloma (ALCYONE): a randomised, open‐label, phase 3 trial. Lancet. 2020;395(10218):132‐141. 31836199 10.1016/S0140-6736(19)32956-3

[15] Facon T , Cook G , Usmani SZ , et al. Daratumumab plus lenalidomide and dexamethasone in transplant‐ineligible newly diagnosed multiple myeloma: frailty subgroup analysis of MAIA. Leukemia. 2022;36(4):1066‐1077. 34974527 10.1038/s41375-021-01488-8 PMC8979809

[16] Bahlis NJ , Corso A , Mugge LO , et al. Benefit of continuous treatment for responders with newly diagnosed multiple myeloma in the randomized FIRST trial. Leukemia. 2017;31(11):2435‐2442. 28373701 10.1038/leu.2017.111 PMC5668494

[17] Gay F , Larocca A , Wijermans P , et al. Complete response correlates with long‐term progression‐free and overall survival in elderly myeloma treated with novel agents: analysis of 1175 patients. Blood. 2011;117(11):3025‐3031. 21228328 10.1182/blood-2010-09-307645

[18] Ludwig H , Bolejack V , Crowley J , et al. Survival and years of life lost in different age cohorts of patients with multiple myeloma. J Clin Oncol. 2010;28(9):1599‐1605. 20177027 10.1200/JCO.2009.25.2114

[19] Larocca A , Dold SM , Zweegman S , et al. Patient‐centered practice in elderly myeloma patients: an overview and consensus from the European myeloma network (EMN). Leukemia. 2018;32(8):1697‐1712. 29880892 10.1038/s41375-018-0142-9

[20] Yao Y , Sui WW , Liao AJ , et al. Comprehensive geriatric assessment in newly diagnosed older myeloma patients: a multicentre, prospective, non‐interventional study. Age Ageing. 2022;51(1):afab211. 34673897 10.1093/ageing/afab211

[21] Zhong YP , Zhang YZ , Liao AJ , Li SX , Tian C , Lu J . Geriatric assessment to predict survival and risk of serious adverse events in elderly newly diagnosed multiple myeloma patients: a multicenter study in China. Chin Med J (Engl). 2017;130(2):130‐134. 28091402 10.4103/0366-6999.197977 PMC5282667

[22] Pawlyn C , Cairns D , Kaiser M , et al. The relative importance of factors predicting outcome for myeloma patients at different ages: results from 3894 patients in the myeloma XI trial. Leukemia. 2020;34(2):604‐612. 31611625 10.1038/s41375-019-0595-5 PMC7214257

